# Clinical and Radiological Characterization of Patients with Immobilizing and Progressive Stress Fractures in Methotrexate Osteopathy

**DOI:** 10.1007/s00223-020-00765-5

**Published:** 2020-10-16

**Authors:** Tim Rolvien, Nico Maximilian Jandl, Julian Stürznickel, Frank Timo Beil, Ina Kötter, Ralf Oheim, Ansgar W. Lohse, Florian Barvencik, Michael Amling

**Affiliations:** 1 Department of Osteology and Biomechanics, University Medical Center Hamburg-Eppendorf, Lottestraße 59, 22529 Hamburg, Germany; 2 Department of Orthopedics, University Medical Center Hamburg-Eppendorf, Martinistraße 52, 20246 Hamburg, Germany; 3 3rd Department of Medicine (Rheumatology), University Medical Center Hamburg-Eppendorf, Martinistraße 52, 20246 Hamburg, Germany; 4 1st Department of Medicine, University Medical Center Hamburg-Eppendorf, Martinistraße 52, 20246 Hamburg, Germany

**Keywords:** Methotrexate, Stress fracture, MTX osteopathy, Cone beam CT, Denosumab, Teriparatide

## Abstract

Methotrexate (MTX) is one of the most commonly prescribed drugs for autoimmune rheumatic diseases. As there is no consensus on its negative effects on bone, the purpose of this investigation was to determine the clinical spectrum of patients with stress fractures due to long-term MTX treatment (i.e., MTX osteopathy). We have retrospectively analyzed data from 34 patients with MTX treatment, severe lower extremity pain and immobilization. MRI scans, bone turnover markers, bone mineral density (DXA) and bone microarchitecture (HR-pQCT) were evaluated. Stress fractures were also imaged with cone beam CT. While the time between clinical onset and diagnosis was prolonged (17.4 ± 8.6 months), the stress fractures had a pathognomonic appearance (i.e., band-/meander-shaped, along the growth plate) and were diagnosed in the distal tibia (53%), the calcaneus (53%), around the knee (62%) and at multiple sites (68%). Skeletal deterioration was expressed by osteoporosis (62%) along with dissociation of low bone formation and increased bone resorption. MTX treatment was discontinued in 27/34 patients, and a combined denosumab–teriparatide treatment initiated. Ten patients re-evaluated at follow-up (2.6 ± 1.5 years) had improved clinically in terms of successful remobilization. Taken together, our findings provide the first in-depth skeletal characterization of patients with pathognomonic stress fractures after long-term MTX treatment.

**Electronic supplementary material:**

The online version of this article (10.1007/s00223-020-00765-5) contains supplementary material, which is available to authorized users.

## Introduction

Methotrexate (MTX) is a folate antagonist that is used in low doses (5–25 mg/week) in the first-line treatment of rheumatoid arthritis (RA), as well as in other inflammatory diseases such as systemic lupus erythematosus (SLE). It acts by inhibiting dihydrofolate reductase, which is an essential factor in DNA and RNA synthesis, but also has far-reaching anti-inflammatory and immunoregulatory effects [1, 2]. While the effects of MTX on bone metabolism have not been completely elucidated, there is both clinical and in vitro evidence for its adverse effects on bone.

MTX osteopathy was first described in 1984 in children who had undergone prolonged maintenance therapy with oral MTX due to acute lymphocytic leukemia (ALL) [3], where distal femoral and tibial fractures with thick dense provisional zones of calcification were detected. In the 1990s, several clinical reports described “MTX osteopathy”, including low bone mass and stress fractures of the distal and proximal tibiae [4–6]. Since then, stress fractures of the proximal tibia have also been described in other patients after long-term MTX use [7, 8]. Inhibitory effects of MTX on osteoblastic bone formation have been detected in human biopsies as well as in murine bone cells [4, 9, 10], and bone mechanical properties were impaired in MTX-treated rats [11]. Although the documented effects of MTX include stimulation of both pro-inflammatory and anti-inflammatory pathways, increased cytokine production may be a possible mechanism for tissue damage in certain conditions such as in MTX osteopathy. In this regard, MTX treatment in cell culture has been linked to a dose-dependent increase in pro-inflammatory cytokines such as IL-1 and IL-6 [12]. At higher doses, MTX use led to increased TNF-α levels and promoted osteoclastogenesis [13]. MTX osteopathy has also been questioned based on the observation that MTX treatment in small patient cohorts and after short-term follow-up did not result in changes in bone turnover or bone density [14], and comparable bone loss compared to other disease-modifying antirheumatic drugs (DMARDs) [15]. Therefore, the clinical relevance of MTX osteopathy remains unclear.

In general, the impairment of bone mineral density and quality as well as the increased risk of fracture have been reported in a variety of rheumatic diseases; however, they are often attributed to inflammatory processes and corticosteroid therapy [16–18]. As there are indications of low bone formation and elevated bone resorption in MTX osteopathy, a combination of anti-resorptive (i.e., denosumab) and osteoanabolic (i.e., teriparatide) therapy might be the most logical treatment option to improve bone strength and recover from stress fractures. We have previously demonstrated recovery from stress fractures in an SLE patient with MTX osteopathy after MTX discontinuation and combined denosumab–teriparatide treatment [19]. Here, we present a clinical characterization of 34 patients suffering from stress fractures after long-term methotrexate use for different underlying rheumatologic diseases and outline their positive response to denosumab and teriparatide treatment in a subset of ten patients.

## Methods

### Subjects

Thirty-four patients with long-term (> 3 years) methotrexate use and stress fractures were included in this study. All patient data were evaluated in a retrospective and anonymized design. Data were analyzed according to the rules of the local ethics committee of the University Medical Center Hamburg-Eppendorf, Germany.

### Skeletal Assessment and Imaging Studies

While magnetic resonance imaging (MRI) was performed for individual stress fracture detection, we also determined the areal bone mineral density (aBMD) using dual-energy X-ray absorptiometry (Lunar iDXA; GE Healthcare; Madison, WI, USA) and bone turnover markers from serum and urine samples in all patients. The measured serum markers included 25-hydroxyvitamin D (25-OH-D_3_), parathyroid hormone (PTH), osteocalcin (Oc), bone-specific alkaline phosphatase (BAP), and deoxypyridinoline cross-links in the urine (DPD).

Furthermore, bone microarchitecture was analyzed in 30/34 patients using high-resolution peripheral quantitative computed tomography (HR-pQCT; XtremeCT, Scanco Medical, Switzerland). In the remaining four patients, HR-pQCT could not be performed due to bilateral distal tibia fractures. Scans were performed in the nonfractured distal tibia following a standardized procedure using the standard in vivo patient evaluation protocol [20, 21]. Specifically, we analyzed the trabecular bone mineral density (Tb.BMD), trabecular number (Tb.N), trabecular thickness (Tb.Th) and cortical bone mineral density (Ct.BMD).

Stress fractures were also imaged in ten patients using cone beam computed tomography (CBCT) working at 90 kV, 40 mAs with a field of view of 16 × 16 ×  13 cm and slice thickness of 0.2 mm (SCS MedSeries H22, Planmed Oy, Helsinki, Finland). CBCT is a novel imaging technique using divergent X-rays that form a cone, which is increasingly used in extremity imaging, though its initial application was in dentistry and maxillofacial surgery [22, 23]. CBCT enables better fracture detection at extremity sites compared to standard radiography [24]. Furthermore, the spatial resolution of CBCT is higher and the radiation dose exposure is lower compared to conventional multislice CT [25].

### Bone Biopsy Studies

We obtained four bone biopsies from individuals with long-term MTX treatment and stress fractures. The first biopsy was obtained from an 81-year-old woman suffering from a stress fracture of the proximal right tibia. The biopsy was obtained from the lateral (unaffected) part of the tibial plateau in the course of total knee arthroplasty (TKA) and compared to a biopsy obtained from a 79-year-old female patient with primary osteoarthritis undergoing TKA. The other three biopsies were obtained from fracture sites as a part of a diagnostic work-up in these patients. While the second biopsy was obtained from the distal tibia of a 64-year-old patient previously reported elsewhere [19], the third and fourth biopsies were taken from the distal tibia and the calcaneus of a 71-year-old woman with RA.

The specimens were fixed in 3.7% formaldehyde, dehydrated, embedded in methyl-methacrylate, and cut on a Microtec rotation microtome (CVT 4060E, Micro Tec, Walldorf, Germany). Afterwards the 5-µm sections were stained with toluidine blue and von Kossa. Histomorphometric analysis was performed according to the ASBMR nomenclature committee [26]. BV/TV, Tb.N, and Tb.Th as well as osteoid volume per bone volume (OV/BV) were evaluated in the von Kossa-stained sections. The osteoblast surface per bone surface (Ob.S/BS) and the osteoclast surface per bone surface (Oc.S/BS) were evaluated from the toluidine blue-stained sections. Quantitative backscattered electron imaging (qBEI) was performed on the first biopsy using a scanning electron microscope (LEO 435 VP; LEO Electron Microscopy Ltd., Cambridge, England) with a backscattered electron detector (Type 202; K.E. Developments Ltd., Cambridge, England), as described previously [27]. The scanning electron microscope was operated at 20 kV and 680 pA at a constant working distance. The acquired images were analyzed using a customized MATLAB (The MathWorks, Inc., Natick, Massachusetts, USA) script. QBEI was used to measure the bone mineral density distribution based on the generated gray values that represent the mean calcium content (mean Ca-Wt%). For both samples, calcium distribution curves were calculated.

### Treatment Intervention and Follow-Up

Ten patients were evaluated at a follow-up visit at 2.6 ± 1.5 years (min. 1 year, max. 5 years). MTX treatment had been discontinued in 8/10 patients in consultation with a rheumatologist, while the MTX dose was reduced in the remaining 2 patients. Seven out of 10 patients were treated with a subcutaneous administration of denosumab 60 mg (Prolia®, Amgen, USA) every 6 months in combination with a daily subcutaneous administration of teriparatide 20 µg (Forsteo®, Eli Lilly, USA). While two patients were not treated with this bone-specific therapy due to individual contraindications, one patient was treated with teriparatide only. The two patients with no bone-specific treatment had discontinued their MTX medication. All patients received 20,000 IE vitamin D3 and 1 g of dietary calcium daily. At follow-up, clinical reexamination and an HR-pQCT scan were performed. The clinical course was evaluated, and patients were asked about subjective changes in mobility and pain (++/+ major/minor improvement, −/− no improvement/worsened). Furthermore, we performed the Timed Up and Go test and compared the achieved time to previously published reference values [28]. The chair rising test (CRT) was performed as described previously [29], and the results were compared to reference data [30].

### Statistical Analysis

All data were evaluated using GraphPad Prism® (GraphPad Software, La Jolla, CA, USA). Data are presented as scatter plots with additional labeling of the mean value ± the standard deviation (SD). After checking for normal distribution, the paired *t*-test was used to compare measurement results from the initial presentation and follow-up. Furthermore, the percent change per year was calculated. *P*-values of 0.05 or less were considered statistically significant.

## Results

### Clinical Characterization

**Fig. 1:**
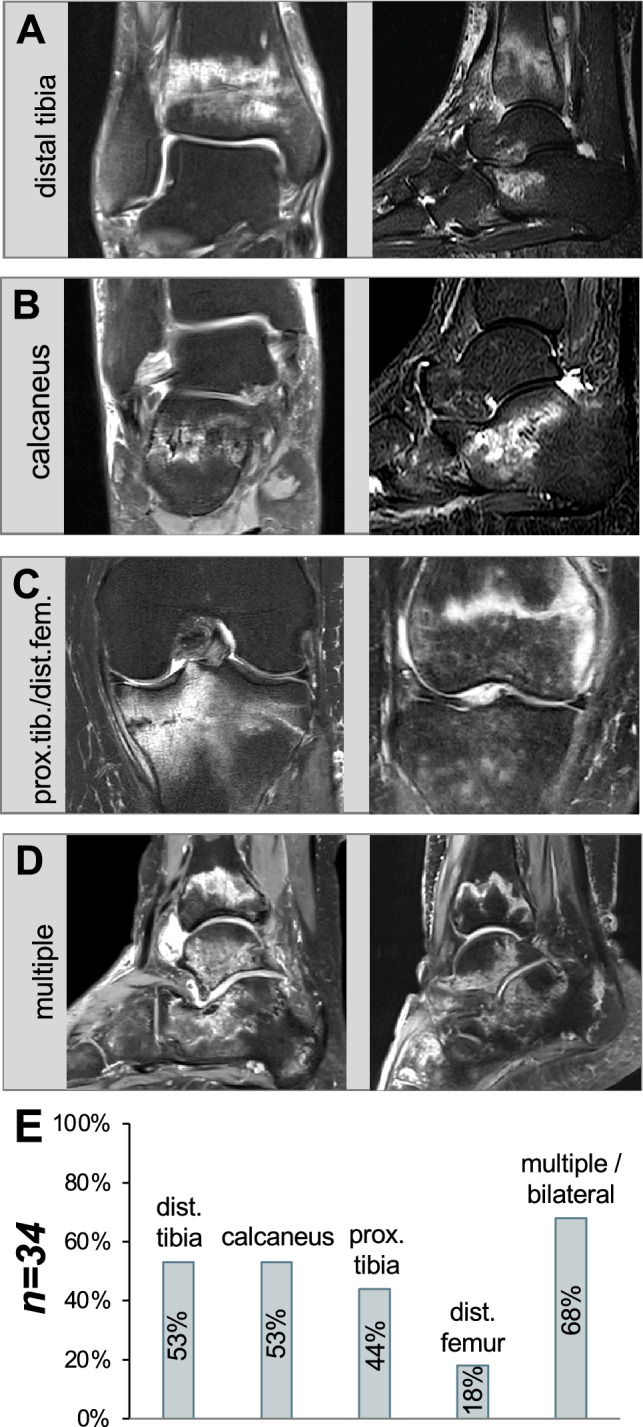
MRI morphology of stress fractures in MTX osteopathy. Band-like stress fractures in proton density (PD)-weighted fat-suppressed (FS) turbo spin echo (TSE) MRI sequences are seen. **a** Distal tibia, coronal and sagittal plane of two different patients. **b** Calcaneus, coronal and sagittal plane of two different patients. **c** Proximal tibia/distal femur, coronal plane. **d** Multiple stress fractures were found in 68% of the patients. **e** Bar graph indicating the regional distribution of stress fractures in *n* = 34 patients (frequency in % for each skeletal site)

A total of 34 patients were evaluated, of whom all had load-dependent pain of the lower limb without adequate trauma in the past. MRI pointed to band- or meander-shaped stress fractures paralleling the former provisional zones of calcification and growth plates in all cases. The most frequent location was the distal tibia (53%) and the calcaneus (53%), followed by the proximal tibia (44%) and the distal femur (18%). In 68% of the patients, we observed stress fractures at multiple and/or bilateral locations (Fig. 1a–e). No association between the time interval of clinical onset and diagnosis and the MTX treatment durations or doses could be detected.

**Fig. 2:**
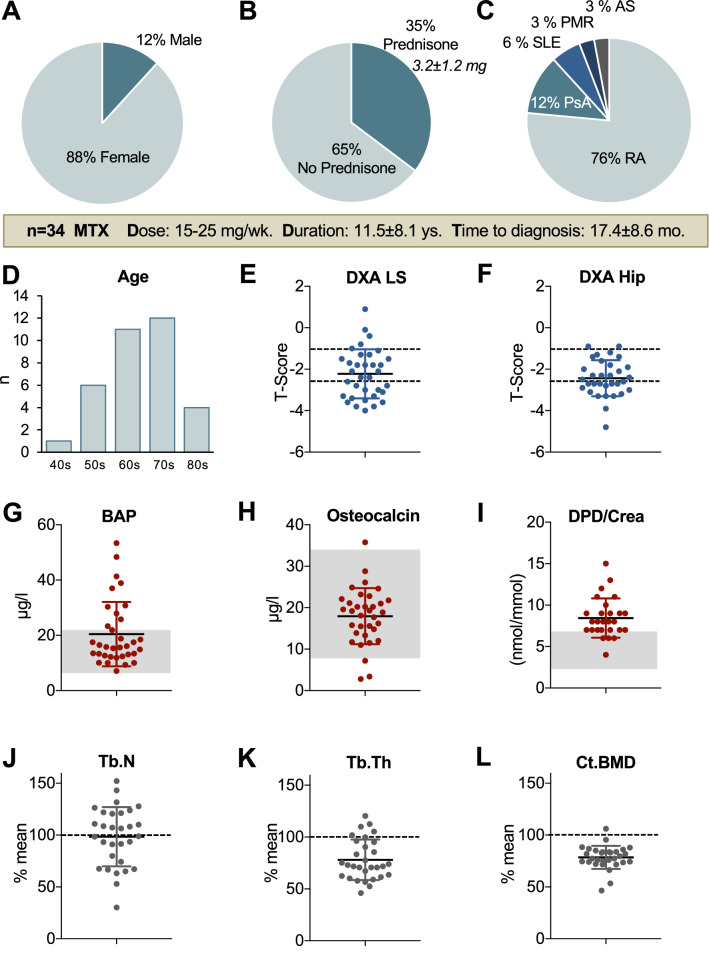
Clinical characteristics and bone mineral density, turnover and microstructure. **a** Eighty-eight percent of the affected patients were female. **b** Prednisone/no prednisone treatment. **c** Distribution of the different rheumatic diseases. **d** Age distribution. **e**, **f** DXA *T*-score at the lumbar spine (LS) and total hip. **g**, **h** Serum bone-specific alkaline phosphatase (BAP) and osteocalcin levels (both bone formation) and **i** urinary deoxypyridinoline (DPD) (bone resorption). Gray boxes indicate reference ranges. **j** Trabecular number (Tb. N), **k** trabecular thickness (Tb. Th) and **l** cortical BMD compared to age- and sex-matched reference data [31]

MTX osteopathy was diagnosed mostly in females (88%, Fig. 2a). The MTX dosage was 18.6 ± 4.9 mg (15–25 mg) weekly. Importantly, a prolonged time between clinical onset and diagnosis of 17.4 ± 8.6 months (5–36 months) was noted. Most patients had a history of corticosteroid treatment; however, upon presentation at our clinic, only 12/34 patients (35.3%) received low-dose oral prednisone (< 5 mg) (Fig. 2b). MTX treatment had been prescribed due to RA (26/34 patients), psoriatic arthritis (4/34 patients), SLE (2/34 patients), polymyalgia rheumatica (1/34 patients) or ankylosing spondylitis (1/34 patients) (Fig. 1c). Only two patients had vertebral fractures. We observed a peak in the occurrence of MTX osteopathy at the age of 70–79 years (Fig. 2d).

A total of 21/34 (61.8%) of the patients were diagnosed with osteoporosis (i.e., BMD *T*-score ≤ − 2.5), and the remaining patients were diagnosed with osteopenia, according to the DXA measurements (Fig. 2e, f). Biochemical bone turnover analyses revealed higher levels in the bone-specific alkaline phosphatase than in the osteocalcin levels compared to reference values (Fig. 2g, h). In 18/32 patients (56%), we detected elevated bone resorption markers in the urine (i.e., DPD cross links) (Fig. 2i). HR-pQCT analysis revealed an almost normal trabecular number in most patients and a more severe reduction of trabecular thickness and cortical BMD in the distal tibia compared to reference values (Fig. 2j–l). While two patients presented with 25-hydroxy-vitamin D3 levels < 20 µg/l, ten patients had 25-OH-D3 levels < 30 µg/l. All patients received oral vitamin D supplementation.

### Cone Beam CT (CBCT) Imaging and Biopsy Findings

**Fig. 3:**
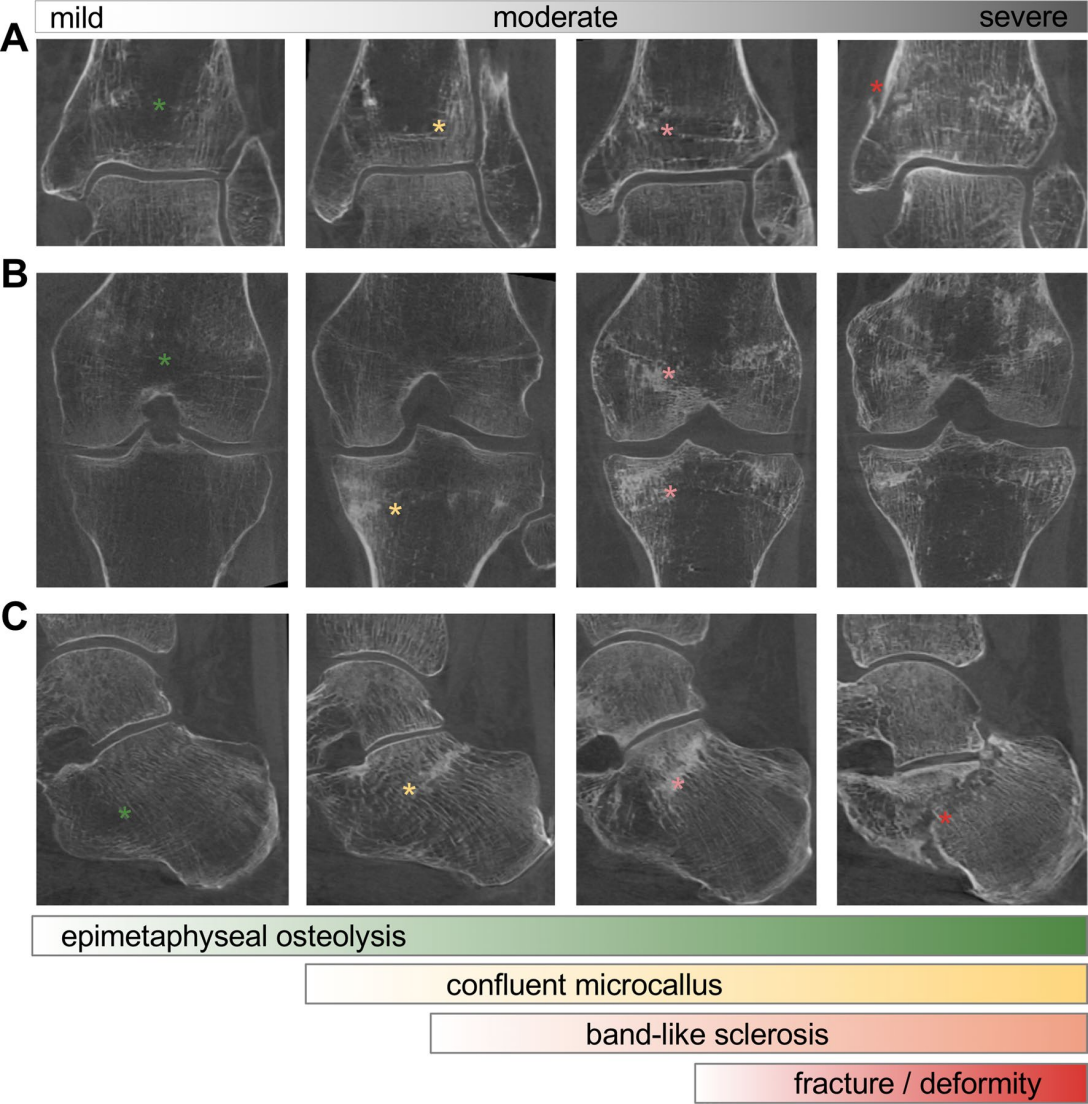
Cone beam CT (CBCT) imaging. Differentiation and classification of different disease severities based on CBCT imaging **a** in the distal tibia, **b** around the knee (distal femur and proximal tibia) and **c** in the calcaneus

CBCT imaging demonstrated osseous alterations around the typical stress fracture localizations (distal tibia, around the knee, calcaneus) (Fig. 3a–c). We were able to determine four stages according to the severity of the morphological alterations. Specific findings included epimetaphyseal osteolysis, followed by confluent microcallus formation and band-like sclerosis along the growth plates. These stress fractures were prone to eventually collapsing, leading to severe fractures and deformities in some cases (Supplementary Fig. 1).

**Fig. 4:**
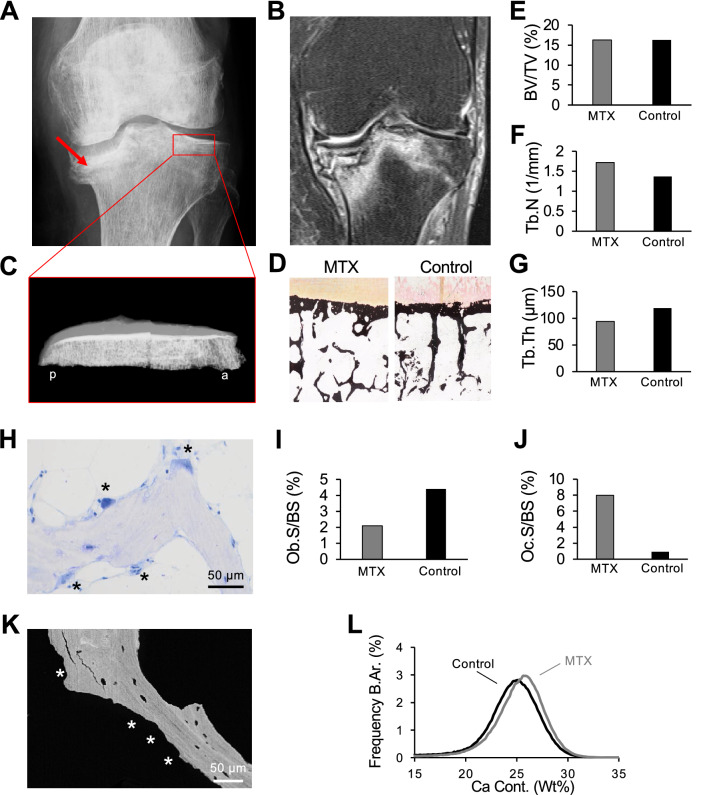
Biopsy studies (81-year-old woman) in the lateral (nonfractured) tibial plateau. **a** Anteroposterior radiograph showing the fracture of the medial tibial plateau (red arrow). **b** Coronal MRI, PD-weighted sequence. **c** Contact radiography (lateral view) of the resected tibial plateau, *a* anterior, *p* posterior. **d** Histological overview, von Kossa staining in the patient and the control. **e**–**g** Quantification of BV/TV, Tb.N and Tb.Th. **h** Visible osteoclasts on the surface of a trabecula, toluidine blue staining. **i**, **j** Osteoblast and osteoclast surface. **k** Image obtained by backscattered electron imaging with eroded surfaces (asterisks). **l** BMDD histograms

**Fig. 5:**
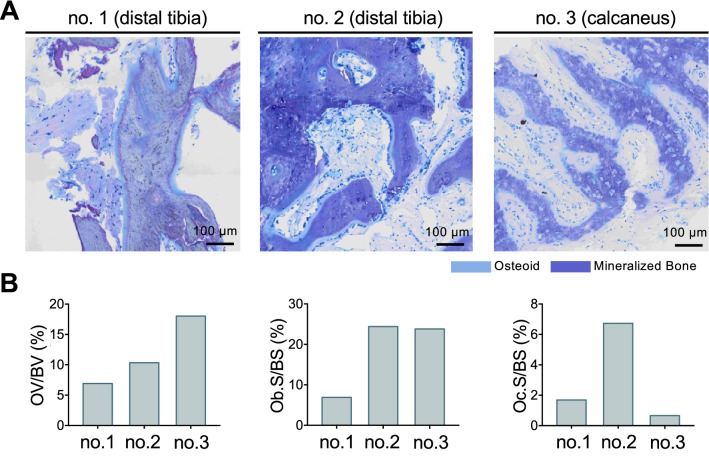
Biopsies from different fracture sites reveal interference with fracture healing with chronic callus formation and osteoidosis but an absence of osteonecrosis. **a** Representative histological images, toluidine blue staining. Left (no. 1): distal tibia (64-year-old woman) showing fracture callus; middle and right (nos. 2, 3): distal tibia and calcaneus (71-year-old woman) with woven bone formation. **b** Histomorphometric quantification in the individual biopsies nos. 1–3 including osteoid volume per bone volume (OV/BV), osteoblast surface per bone surface (Ob.S/BS) and osteoclast surface per bone surface (Oc.S/BS)

To further characterize the skeletal changes on a microscopic level, a biopsy was obtained from the lateral (nonfractured) part of the tibial plateau. In this patient, the stress fracture of the medial tibial plateau was already visible on conventional radiography (Fig. 4a) and later confirmed by MRI (Fig. 4b). The tibial plateau was subsequently imaged by contact radiography (Fig. 4c). Histological quantification was performed in comparison with an age-matched control (Fig. 4d). The tibial bone microstructure of the patient with MTX osteopathy was characterized by an unchanged BV/TV and a higher trabecular number but a lower trabecular thickness (Fig. 4e–g). Furthermore, a low number of osteoblasts but a high number of osteoclasts were detected, leading to a lower osteoblast surface and a higher osteoclast surface compared to those of the control (Fig. 4h–j). Backscattered electron imaging confirmed highly prevalent eroded surfaces but an overall similar bone mineral density distribution (Fig. 4k, l). Additional histomorphometric analysis of three fracture biopsies from the distal tibiae and the calcaneus revealed the presence of fracture calluses and woven bone but no detection of osteonecrosis. This was associated with a generally high bone turnover (Fig. 5).

### Treatment Outcome and Follow-Up

MTX treatment was discontinued or replaced in 27/34 patients, while the remaining seven patients underwent MTX dose reduction. The discontinuation of MTX, in consultation with an expert rheumatologist, was well tolerated. MTX was replaced by azathioprine, low-dose glucocorticoids or monoclonal antibodies (e.g., IL-17). Moreover, a bone-specific therapy consisting of denosumab, teriparatide or combined denosumab–teriparatide was initiated on the basis of the risk profiles and pre-existing comorbidities.

**Fig. 6:**
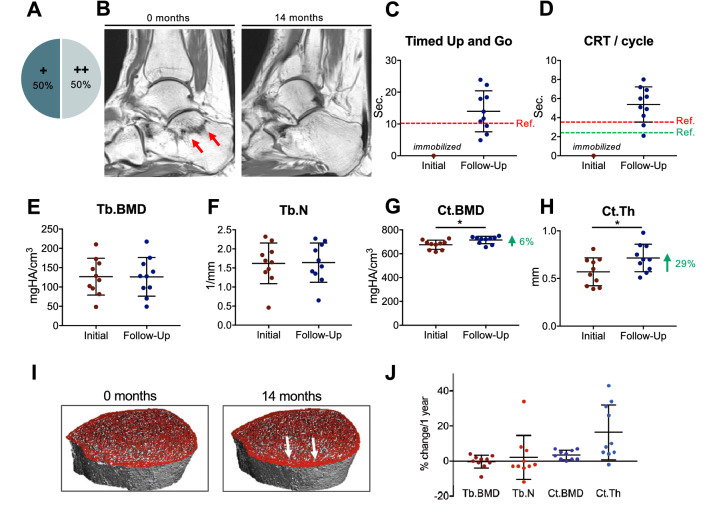
Follow-up and treatment response. **a** Subjective clinical course (++ major improvement, + minor improvement, − no improvement in mobility and pain). **b** MRI, T1-weighted, sagittal sequence. **c** Individual results for the Timed Up and Go test at follow-up. **d** Chair rising test (CRT). **e**–**j** HR-pQCT at initial presentation and follow-up (distal tibia for all panels). **e** Trabecular BMD. **f** Trabecular number. **g** Cortical BMD. **h** Cortical thickness. **i** HR-pQCT image at 0 and 14 months indicating increasing cortical thickness (white arrows). **j** HR-pQCT changes converted to %-change/1 year

Follow-up examinations in ten patients (8/10 MTX discontinuation) revealed that no further stress fractures had occurred, while treatment modifications were well tolerated and overall mobility was improved. Most patients showed an adequate increase or at least stable BMD values in the lumbar spine and hip (Table 1). Five of ten patients reported major improvements regarding mobility and pain levels, while the other five patients reported minor improvements (Fig. 6a). Of the 5/10 patients with a major improvement, all had discontinued their MTX medication. The two patients who continued MTX had only minor clinical improvements and showed no increase in BMD. Although MRI follow-up could not be performed in all patients, healing of stress fractures was observed in individual patients who underwent follow-up MRI (Fig. 6b). Successful remobilization and return to daily activities were achieved in all cases. Timed Up and Go testing confirmed the regained mobility, although the reference of 10 s was not reached in most patients (Fig. 6c). The CRT indicated slow but possible rises from a chair, while both tests were not possible in any of the patients at the initial presentation (Fig. 6d). The analysis of bone microarchitecture in the tibia using HR-pQCT revealed constant trabecular parameters and marked improvements in cortical parameters at follow-up (Fig. 6e–j, Supplementary Tab. 1 + 2).

**Table 1:** BMD and corresponding *T*-scores assessed by DXA in the lumbar spine (LS) and hip of ten patients with follow-up measurements

Pat.	Time interval (yr.)	MTX	Specific therapy	CC	BMD LS			*T*-score LS			BMD hip			*T*-score hip		
					Initial	Follow-up	Change (%)	Initial	Follow-up	Change (SD)	Initial	Follow -up	Change (%)	Initial	Follow -up	Change (SD)
1	1.5	DIS	DATA	++	1.027	1.012	− 1.5	− 1.3	− 1.3	± 0.0	0.810	0.831	+ 2.6	− 1.4	− 1.2	+ 0.2
2	2.3	CON	DATA	+	n/a	n/a	n/a	n/a	n/a	n/a	0.671	0.660	− 1.6	− 3.2	− 3.6	− 0.4
3	4.0	DIS	DATA	+	0.869	0.957	+ 10.1	− 2.8	− 2.0	+ 0.8	0.675	0.686	+ 1.6	− 2.7	− 2.6	+ 0.1
4	4.0	DIS	DATA	++	1.121	1.054	− 6.0	− 0.8	− 0.9	− 0.1	0.689	0.717	+ 4.1	− 2.6	− 2.2	+ 0.4
5	3.8	DIS	DATA	++	0.709	0.788	+ 11.1	− 3.8	− 2.4	+ 1.4	0.717	0.726	+ 1.3	− 2.4	0.1	+ 2.5
6	1.6	DIS	no	++	0.713	0.764	+ 7.2	− 4.0	− 3.6	+ 0.4	0.609	0.580	− 4.8	− 3.3	− 3.5	− 0.2
7	1.0	DIS	DATA	++	0.950	0.962	+ 1.3	− 1.8	− 1.7	+ 0.1	0.784	0.797	+ 1.7	− 1.6	− 1.5	+ 0.1
8	1.6	DIS	no	+	0.743	0.785	+ 5.7	− 3.5	− 3.3	+ 0.2	0.660	0.689	+ 4.4	− 2.7	− 2.4	+ 0.3
9	1.0	DIS	DATA	+	1.028	1.078	+ 4.9	− 1.1	− 0.7	+ 0.4	0.779	0.864	+ 10.9	− 1.8	− 1.1	+ 0.7
10	5.0	CON	TPT → D’mab	+	0.988	0.961	− 2.7	− 1.8	− 1.8	± 0.0	n/a	n/a	n/a	n/a	n/a	n/a

Changes are presented as percent (%) or standard deviation (SD)

*Yr.* years, *DIS* discontinued, *CON* continued, *DATA* denosumab and teriparatide, *D’mab* denosumab, *TPT* teriparatide, *CC* clinical course (++/+ major/minor clinical improvement), *n/a* not available due to previous orthopedic surgery such as spondylodesis or hip replacement

## Discussion

While stress fractures in patients with long-term MTX use have been repeatedly described, detailed knowledge on the underlying skeletal alterations in a larger patient collective was not available to date. From a clinical perspective, our results suggest that long-term MTX treatment may have negative effects on bone metabolism and quality in certain patient groups, which was expressed by stress fractures with a unique band- or meander-shaped appearance along the growth plate. Indeed, our MRI-based morphological characterization therefore corresponds well to the first reports on MTX osteopathy [3, 4, 6]. In other words, this comprehensive imaging analysis illustrates that the detected uniquely shaped stress fractures constitute the hallmark of MTX osteopathy. Even in different rheumatic diseases, this typical clinical and radiological picture was detected, emphasizing the concept of significant MTX side effects rather than concomitant phenomena of the underlying diseases. At the same time, stress fractures due to other reasons including overuse, osteomalacia (e.g., calcium malabsorption, phosphate wasting) or inflammatory conditions are typically not characterized by a meander-shaped morphology along the growth plates.

The skeletal alterations were subsequently characterized by CBCT, where a consistent radiographic pattern of epimetaphyseal osteolysis and band-like sclerosis (i.e., microcallus) was found. In association with this pathognomonic imaging appearance detected by MRI and CBCT, we observed a consistent bone microstructure and turnover pattern. Namely, the skeletal deterioration was further characterized by HR-pQCT, where pronounced trabecular thinning was detected. Bone turnover was often characterized by a combination of high bone-specific alkaline phosphatase levels, low osteocalcin levels indicating low bone formation, and elevated bone resorption parameters (i.e., urinary DPD cross links). This dissociation between low bone formation and high bone resorption was recapitulated in a bone biopsy obtained from a patient with MTX osteopathy, while three biopsies from fracture sites indicated interference with fracture healing but an absence of osteonecrosis. In sum, the detected bone turnover dissociation is most likely a major contributing factor for the detected long-term skeletal complications.

Based on our findings, we suggest that any symptomatic patient with lower extremity pain, loss of mobility and long-term MTX treatment should be rigorously screened for stress fractures and skeletal status (i.e., osteoporosis) using MRI, DXA and laboratory analyses. CBCT represents a valuable tool to further image these osseous alterations at a high resolution while simultaneously reducing radiation exposure [22]. While the correct diagnosis can help to ensure the best medical care in terms of appropriate treatment, there is a risk that stress fractures will progress towards extensive bone and joint destruction (i.e., collapse) if not treated properly and MTX is continued in these patients (Supplementary Fig. 1). Although we do understand that a discontinuation of MTX might not be feasible in all patients with different rheumatic inflammatory diseases, MTX discontinuation should always be considered in patients with MTX osteopathy.

As the diagnosed stress fractures were accompanied by osteoporosis or osteopenia, according to DXA, and a dissociation of low bone formation and elevated bone resorption in most cases, we treated most our patients with both denosumab (anti-resorptive) and teriparatide (osteoanabolic) according to the previously published DATA study [32]. We additionally guaranteed sufficient 25-OH-D3 levels, as this was found to be beneficial for reducing the risk of stress fractures [33]. In combination with a discontinuation of MTX treatment, the analyzed subset of ten patients recovered from stress fractures and regained their mobility, which was associated with increased BMD levels and improved cortical microstructure in most patients. Close rheumatologic monitoring was carried out to avoid flares of the underlying autoimmune disease.

The denosumab–teriparatide combination therapy was previously shown to lead to the highest increases in BMD [32] before the sclerostin antibody romosozumab entered clinical research [34]. However, it is currently not known whether these findings can be applied to special patient groups such as RA patients with MTX osteopathy. In rats with MTX-induced osteopathy, anti-resorptive treatment was found to be effective [11]. The addition of denosumab to MTX has also been found to be an effective treatment option in the prevention of further joint destruction in patients with RA [35].

We are aware that our study only offers correlative evidence regarding MTX osteopathy as a clinical entity that is described by pathognomonic and nonself-limiting stress fractures. Our results do not provide sufficient information in terms of causality and stress fracture prevalence due to long-term MTX treatment, and future studies will have to elaborate on the epidemiologic aspects of this burden. Our data also do not allow us to determine whether MTX discontinuation or the initiated bone-specific therapy is more effective regarding the achieved clinical improvements. It is interesting to note that from the ten patients analyzed at follow-up, the two patients who continued MTX showed minor clinical improvements and no increase in BMD. Finally, other conditions, such as inflammation, corticosteroid use or postmenopausal status, also promote an impairment in bone health. Nonetheless, several studies have linked MTX to deteriorated bone quality. Reduced BMD was observed with MTX use compared to no MTX use among patients with RA [36]. In a prospective trial of 329 RA patients, a negative annual BMD change was significantly associated with MTX dosage [37]. Moreover, even in several other reported cases of patients with RA and stress fractures, most of them had previously undergone long-term MTX therapy [38–40]. On the other hand, MTX was associated with neither improved nor worsened bone microstructure in patients with PsA, while DMARD use (such as TNFalpha inhibitors or IL-17A antibodies) led to improved microstructure [41]. In general, it remains a major issue to develop optimal drug strategies for treating bone problems in patients with inflammatory diseases [42].

Taken together, we have provided clinical evidence of the potential negative effects of long-term MTX use on bone quality, associated with peculiar stress fractures that can be positively influenced by a discontinuation of MTX together with the initiation of a combined denosumab and teriparatide treatment. Whether the observed improvements in stress fractures and changes in quality translate into reductions in stress fracture risk over the long term remains to be evaluated in larger series.

## Electronic supplementary material

Below is the link to the electronic supplementary material.

Supplementary file1 (PDF 1308 kb)
