# Supplementary material for: Clinical and Radiological Characterization of Patients with Immobilizing and Progressive Stress Fractures in Methotrexate Osteopathy
Source: Calcif Tissue Int. 2020 Oct 16;108(2):219–30. doi: 10.1007/s00223-020-00765-5 (PMC7819927; doi:10.1007/s00223-020-00765-5)
Supplement: Supplementary file 1 — Supplementary file1 (PDF 1308 kb) [file 223_2020_765_MOESM1_ESM.pdf]

Supplementary Material

Clinical and radiological characterization of patients with immobilizing and progressive stress fractures in methotrexate osteopathy

Tim Rolvien, Nico Maximilian Jandl, Julian Stürznickel, Frank Timo Beil, Ina Kötter, Ralf Oheim, Ansgar W Lohse, Florian Barvencik and Michael Amling

A B C

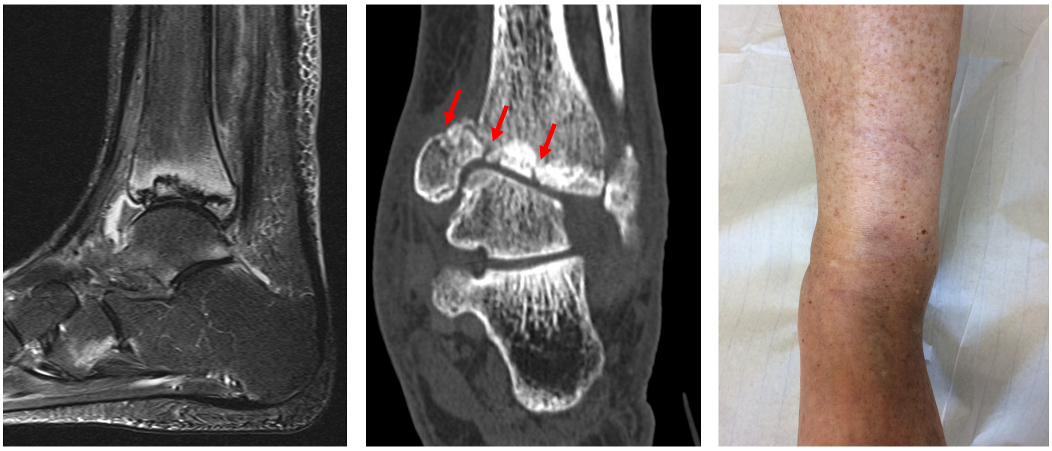

Supplementary Fig. 1: Progression of a distal tibia stress fracture in a 73-year-old female patient towards total collapse and subluxation of the upper ankle joint. (A) Sagittal MRI, turbo inversion recovery magnitude (TIRM) sequence. (B) Coronal CT reformat demonstrating the multi-fragment fracture. (C) Note the dislocation of the upper ankle joint that could already be seen at the initial clinical examination.

Supplementary Tab. 1: HR-pQCT data of patients with available follow-up measurement in the distal radius with Tb.BMD (HA/cm^3), Tb.N (1/mm), Ct.BMD (HA/cm^3) and Ct.Th (mm) and percentage (%) of reference values published by Burt et al. (2016). Changes of absolute HR-pQCT values between initial and follow-up measurement presented in percentage (%).

Pat.	time interval (yr.)	MTX	specific therapy	CC	Tb.BMD (HA/cm^3)			Tb.N (1/mm)			Ct.BMD (HA/cm^3)			Ct.Th (mm)		
					initial	*follow up*	*change (%)*	*initial*	*follow up*	*change (%)*	*initial*	*follow up*	*change (%)*	*initial*	*follow up*	*change (%)*
1	1.5	DIS	DATA	++	149.4 (103%)	152.3 (105%)	+ 1.9	2.09 (128%)	2.01 (123%)	- 3.8	715.3 (79%)	753.7 (83%)	+ 5.4	0.50 (49%)	0.60 (72%)	+ 22.4
2	2.3	CON	DATA	+	122.1 (64%)	129.6 (71%)	+ 6.1	1.37 (74%)	1.60 (90%)	+ 16.8	697.8 (75%)	707.3 (79%)	+ 1.4	0.50 (49%)	0.63 (65%)	+ 26.0
3	4.0	DIS	DATA	+	129.2 (85%)	125.1 (82%)	- 3.2	1.51 (88%)	1.49 (87%)	- 1.3	742.1 (76%)	750.5 (77%)	+ 1.1	0.55 (60%)	0.59 (65%)	+ 7.3
4	4.0	DIS	DATA	++	174.6 (116%)	164.9 (113%)	- 5.6	1.44 (86%)	1.29 (79%)	- 10.4	719.4 (76%)	795.7 (88%)	+ 10.6	0.68 (76%)	0.98 (117%)	+ 44.1
5	3.8	DIS	DATA	++	113.1 (78%)	119.5 82%)	+5.7	1.80 (110%)	1.93 (118%)	+ 7.2	757.3 (83%)	760.2 (84%)	+ 0.4	0.56 (67%)	0.54 (65%)	- 3.6
6	1.6	DIS	no	++	75.8 (52%)	86.0 (59%)	+ 13.5	1.16 (71%)	1.39 (85%)	+ 19.8	826.1 (91%)	812.1 (89%)	- 1.7	0.71 (85%)	0.64 (77%)	- 9.9
7	1.0	DIS	DATA	++	109.0 (75%)	119.4 (82%)	+ 9.5	1.15 (71%)	1.23 (75%)	+ 7.0	722.7 (80%)	732.6 (81%)	+ 1.4	0.40 (48%)	0.43 (51%)	+ 7.5
8	1.6	DIS	no	+	130.5 (87%)	121.9 (81%)	- 6.6	1.47 (87%)	1.03 (61%)	- 29.9	768.4 (81%)	803.7 (85%)	+ 4.6	0.66 (74%)	0.78 (88%)	+ 18.2
9	1.0	DIS	DATA	+	140.2 (93%)	141.1 (94%)	+ 0.6	1.92 (114%)	1.97 (117%)	+ 2.6	857.0 (91%)	860.5 (91%)	+ 0.4	0.84 (94%)	0.85 (95%)	+ 1.2
10	5.0	CON	TPT → D‘mab	+	70.6 (49%)	86.4 (65%)	+ 22.4	1.16 (71%)	1.40 (89%)	+ 20.7	743.0 (82%)	580.7 (66%)	- 21.8	0.46 (55%)	0.28 (38%)	- 39.1

Yr.: years, DIS: discontinued, CON: continued, DATA: denosumab and teriparatide, TPT: teriparatide, CC: Clinical course (++/+ major/minor improvement).

Supplementary Tab. 2: HR-pQCT data of patients with available follow-up measurement in the distal tibia with Tb.BMD (HA/cm^3), Tb.N (1/mm), Ct.BMD (HA/cm^3) and Ct.Th (mm) and percentage (%) of reference values published by Burt et al. (2016). Changes of absolute HR-pQCT values between initial and follow-up measurement presented in percentage (%).

Pat.	time interval (yr.)	MTX	specific therapy	CC	Tb.BMD (HA/cm^3)			Tb.N (1/mm)			Ct.BMD (HA/cm^3)			Ct.Th (mm)		
					initial	*follow up*	*change (%)*	*initial*	*follow up*	*change (%)*	*initial*	*follow up*	*change (%)*	*initial*	*follow up*	*change (%)*
1	1.5	DIS	DATA	++	172.6 (107%)	175.5 (108%)	+ 1.7	2.32 (152%)	2.21 (145%)	- 4.7	629.1 (76%)	691.7 (84%)	+ 10.0	0.40 (37%)	0.66 (61%)	+ 65.0
2	2.3	CON	DATA	+	122.9 (68%)	139.7 (74%)	+ 8.5	2.11 (106%)	2.11 (121%)	+ 14.7	457.6 (73%)	726.2 (85%)	+ 15.4	0.42 (31%)	0.75 (56%)	+ 78.6
3	4.0	DIS	DATA	+	146.7 (91%)	128.3 (80%)	- 12.5	1.47 (93%)	1.35 (86%)	- 8.2	693.7 (75%)	732.7 (79%)	+ 5.6	0.70 (57%)	0.84 (68%)	+ 20.0
4	4.0	DIS	DATA	++	117.9 (72%)	127.6 (79%)	+ 8.2	1.92 (124%)	1.70 (111%)	- 11.5	718.5 (82%)	740.6 (90%)	+ 3.1	0.60 (51%)	0.70 (65%)	+ 16.7
5	3.8	DIS	DATA	++	102.4 (63%)	98.9 (61%)	- 3.4	1.39 (91%)	1.41 (92%)	+ 1.4	692.6 (84%)	723.3 (87%)	+ 4.4	0.54 (50%)	0.70 (65%)	+ 29.6
6	1.6	DIS	no	++	96.4 (60%)	97.5 (60%)	+ 1.1	1.65 (108%)	1.54 (101%)	- 6.7	676.6 (82%)	720.9 (87%)	+ 6.5	0.48 (45%)	0.56 (52%)	+ 16.7
7	1.0	DIS	DATA	++	210.4 (130.0 %)	217.4 (134%)	+ 3.3	1.69 (111%)	2.27 (145%)	+ 34.3	615.5 (74%)	655.3 (79%)	+ 6.5	0.39 (36%)	0.51 (47%)	+ 30.8
8	1.6	DIS	no	+	81.5 (50%)	70.3 (43%)	- 13.7	1.24 (80%)	1.19 (77%)	- 4.0	680.9 (78%)	735.3 (84%)	+ 8.0	0.69 (59%)	0.98 (84%)	+ 42.0
9	1.0	DIS	DATA	+	161.3 (99%)	159.5 (98%)	- 1.1	2.22 (143%)	1.96 (126%)	- 11.7	728.8 (83%)	750.1 (85%)	+ 2.9	0.81 (69%)	0.85 (72%)	+ 4.9
10	5.0	CON	TPT → D‘mab	+	48.5 (30%)	49.1 (30%)	+ 1.2	0.46 (30%)	0.65 (43%)	+ 41.3	686.6 (83%)	672.9 (81%)	- 2.0	0.67 (62%)	0.60 (56%)	- 10.4

Yr.: years, DIS: discontinued, CON: continued, DATA: denosumab and teriparatide, TPT: teriparatide, CC: Clinical course (++/+ major/minor improvement).
